# Methods of Patient Warming during Abdominal Surgery

**DOI:** 10.1371/journal.pone.0039622

**Published:** 2012-07-11

**Authors:** Li Shao, Hong Zheng, Feng-Ju Jia, Hui-Qin Wang, Li Liu, Qi Sun, Meng-Ying An, Xiu-Hua Zhang, Hao Wen

**Affiliations:** 1 Department of Operation Room, The First Teaching Hospital of Xinjiang Medical University, Urumqi, Xinjiang Uygur Autonomous Region, China; 2 Department of Surgery, The First Teaching Hospital of Xinjiang Medical University, Urumqi, Xinjiang Uygur Autonomous Region, China; 3 Department of Anesthesiology, The First Teaching Hospital of Xinjiang Medical University, Urumqi, Xinjiang Uygur Autonomous Region, China; 4 Department of Statistic Counseling, The First Teaching Hospital of Xinjiang Medical University, Urumqi, Xinjiang Uygur Autonomous Region, China; University of Colorado, United States of America

## Abstract

**Background:**

Keeping abdominal surgery patients warm is common and warming methods are needed in power outages during natural disasters. We aimed to evaluate the efficacy of low-cost, low-power warming methods for maintaining normothermia in abdominal surgery patients.

**Methods:**

Patients (n = 160) scheduled for elective abdominal surgery were included in this prospective clinical study. Five warming methods were applied: heated blood transfusion/fluid infusion vs. unheated; wrapping patients vs. not wrapping; applying moist dressings, heated or not; surgical field rinse heated or not; and applying heating blankets or not. Patients’ nasopharyngeal and rectal temperatures were recorded to evaluate warming efficacy. Significant differences were found in mean temperatures of warmed patients compared to those not warmed.

**Results:**

When we compared temperatures of abdominal surgery patient groups receiving three specific warming methods with temperatures of control groups not receiving these methods, significant differences were revealed in temperatures maintained during the surgeries between the warmed groups and controls.

**Discussion:**

The value of maintaining normothermia in patients undergoing abdominal surgery under general anesthesia is accepted. Three effective economical and practically applicable warming methods are combined body wrapping and heating blanket; combined body wrapping, heated moist dressings, and heating blanket; combined body wrapping, heated moist dressings, and warmed surgical rinse fluid, with or without heating blanket. These methods are practically applicable when low-cost method is indeed needed.

## Introduction

In recent years, core body temperature has been considered one of the basic measurements in monitoring patients undergoing general anesthesia. As early as the mid-1990s, observers reported hypothermia in as many as 60% of patients during surgery, with 30% of patients having a core body temperature below 35°C [1]. As a result, complications such as ventricular tachycardia, hypertension, and increased risk of infection associated with intra- and perioperative hypothermia have come to the attention of surgeons and anesthesiologists [2], and various methods of patient warming have been promoted for clinical use to lower the risk of hypothermia associated with administering general anesthesia.

During natural disasters such as earthquakes, tsunami or major flooding, power is generally lost and alternative methods are available, including body wraps and the use of heated moist dressings as well as warmed fluids and blood transfusions and the use of heated blankets. Heating is an option with commonly used practices such as infusion of fluids, blood transfusion, and the application of body wraps, dressings, and blankets. Combinations of these warming methods may be feasible.

The purpose of the present research was to evaluate low-cost, low- or no-power, and readily available alternative warming methods for maintaining normothermia in abdominal surgery patients.

## Methods

### Patient Selection

The present study is a prospective study conducted in the surgical center of the First Hospital of Xinjiang Medical University, Xinjian, China. One hundred sixty patients who scheduled for elective abdominal surgery between October 2009 and May 2010 were selected. Inclusion criteria were as follows: patients between the ages of 18 and 60 with an ASA score of I or II [3]; three days of preoperative temperature within the normal range; procedure conducted under combined intravenous and inhalation anesthesia; patient in supine operative position; procedure not done *per anum*; patient able to tolerate placement of temperature sensor in nasopharynx and rectum; operation time limited to within 5 hours. Exclusion criteria were as follows: presence of systemic metabolic disease; presence of infection or abnormal body temperature three days before operation; hypothermia or shock induced by surgery; surgery interrupted by need to obtain frozen section; change in surgical method due to inconsistency between preoperative and intraoperative diagnoses; cessation or intensification of warming measures due to hyperthermia or hypothermia. Random sampling was applied to select patients for groups receiving various warming methods during surgery.

The study was approved by the ethics committee of the First Affiliated Hospital of Xinjiang Medical University. All patients who participated in the study signed informed consent forms indicating that they knew of the existence of the study, understood its purpose, and agreed to participate.

### Data Collection

Data were collected for five factors (methods of warming) on two levels, one level involving heating or specific application and one without heating or specific application. Five factors were configured as follows: blood transfusions and fluid infusions were either heated or not; patients’ bodies were either wrapped or not; moist dressings were heated or not; surgical field rinse was either heated or not; and heating blankets were either used or not. According to the permutations of the factors (2^5^), a total of 32 combinations were set, and each group received specific combinations repeated 5 times for the total number of 160 patients (Table 1 and 2). The present study used random sampling of patients to determine groups receiving specific warming methods and a double-blind design. The double blind was carried out by having one researcher seal each envelope containing warming instructions and then having the envelope opened by a second researcher, with the operation and warming method conducted according to the instructions in the envelope.

**Table 1:** Array of the 32 groups of patients.

			D1		D2	
			E1	E2	E1	E2
A1	B1	C1	A1B1C1D1E1	A1B1C1D1E2	A1B1C1D2E1	A1B1C1D2E2
		C2	A1B1C2D1E1	A1B1C2D1E2	A1B1C2D2E1	A1B1C2D2E2
	B2	C1	A1B2C1D1E1	A1B2C1D1E2	A1B2C1D2E1	A1B2C1D2E2
		C2	A1B2C2D1E1	A1B2C2D1E2	A1B2C2D2E1	A1B2C2D2E2
A2	B1	C1	A2B1C1D1E1	A2B1C1D1E2	A2B1C1D2E1	A2B1C1D2E2
		C2	A2B1C2D1E1	A2B1C2D1E2	A2B1C2D2E1	A2B1C2D2E2
	B2	C1	A2B2C1D1E1	A2B2C1D1E2	A2B2C1D2E1	A2B2C1D2E2
		C2	A2B2C2D1E1	A2B2C2D1E2	A2B2C2D2E1	A2B2C2D2E2

Key: A1: Bood transfusion and fluid infusion not heated. A2: Blood transfusion and fluid infusion heated. B1: Patient’s body not wrapped. B2: Patient’s body wrapped. C1: Moist dressings not heated. C2: Moist dressings heated. D1: Surgical field rinse not heated. D2: Surgical field rinse heated. E1: Heating blanket not used. E2: Heating blanket used.

**Table 2:** Factors and levels.

Group	Factors and levels					Subject number				
1	A1,	B1,	C1,	D1	E1	**27**	**37**	**87**	**97**	**139**
2	A1,	B1,	C1,	D1	E2	**15**	**36**	**79**	**100**	**152**
3	A1,	B1,	C1,	D2	E1	**13**	**63**	**95**	**123**	**136**
4	A1,	B1,	C1,	D2	E2	**7**	**40**	**88**	**121**	**154**
5	A1,	B1,	C2,	D1	E1	**32**	**51**	**67**	**119**	**135**
6	A1,	B1,	C2,	D1	E2	**4**	**53**	**75**	**128**	**146**
7	A1,	B1,	C2,	D2	E1	**18**	**39**	**85**	**106**	**159**
8	A1,	B1,	C2,	D2	E2	**12**	**35**	**82**	**105**	**153**
9	A1,	B2,	C1,	D1	E1	**30**	**52**	**92**	**122**	**157**
10	A1,	B2,	C1,	D1	E2	**3**	**50**	**73**	**126**	**143**
11	A1,	B2,	C1,	D2	E1	**5**	**57**	**86**	**116**	**158**
12	A1,	B2,	C1,	D2	E2	**11**	**45**	**66**	**127**	**142**
13	A1,	B2,	C2,	D1	E1	**23**	**44**	**90**	**107**	**129**
14	A1,	B2,	C2,	D1	E2	**26**	**55**	**81**	**120**	**138**
15	A1,	B2,	C2,	D2	E1	**2**	**38**	**74**	**110**	**160**
16	A1,	B2,	C2,	D2	E2	**16**	**60**	**76**	**98**	**155**
17	A2,	B1,	C1,	D1	E1	**22**	**48**	**68**	**111**	**130**
18	A2,	B1,	C1,	D1	E2	**24**	**43**	**80**	**113**	**151**
19	A2,	B1,	C1,	D2	E1	**8**	**62**	**70**	**112**	**131**
20	A2,	B1,	C1,	D2	E2	**28**	**46**	**84**	**114**	**144**
21	A2,	B1,	C2,	D1	E1	**21**	**58**	**91**	**103**	**149**
22	A2,	B1,	C2,	D1	E2	**1**	**54**	**69**	**108**	**150**
23	A2,	B1,	C2,	D2	E1	**29**	**33**	**94**	**118**	**148**
24	A2,	B1,	C2,	D2	E2	**19**	**61**	**93**	**104**	**132**
25	A2,	B2,	C1,	D1	E1	**9**	**41**	**78**	**99**	**147**
26	A2,	B2,	C1,	D1	E2	**20**	**59**	**83**	**102**	**145**
27	A2,	B2,	C1,	D2	E1	**17**	**49**	**77**	**125**	**140**
28	A2,	B2,	C1,	D2	E2	**10**	**47**	**89**	**117**	**133**
29	A2,	B2,	C2,	D1	E1	**25**	**64**	**72**	**101**	**141**
30	A2,	B2,	C2,	D1	E2	**31**	**42**	**96**	**109**	**134**
31	A2,	B2,	C2,	D2	E1	**14**	**34**	**71**	**115**	**156**
32	A2,	B2,	C2,	D2	E2	**6**	**56**	**65**	**124**	**137**

The average normal environmental temperature of a patient in the supine position is 23°C. For this study, the core body temperature was measured by rectal temperature, which reflects the temperature of major internal organs such as the liver while the nasopharyngeal temperature reflects the temperature of the brain.

The five methods of warming were carried out as follows: 1) Blood and fluid warming: a MIR-162 electric incubator (Sanyo, Osaka, Japan) was used to warm fluid to 37°C before use; the 37°C fluid was then infused into the patient. Banked blood for transfusion was warmed to 37°C by means of a DK series water bath (Shanghai Jinghong Laboratory Instrument Co., Ltd., Shanghai, China) for half an hour. 2) Body wrap: the patient’s limbs were wrapped from the lower third of the thighs to the feet; the neck and shoulders were also wrapped using lightweight pads that were not prewarmed. 3) Warmed moist dressing: the moist dressings were made of Xinjie brand surgical gauze (Kangqiang Medical Equipment Co., Urumqi, Xinjiang Uyghur Autonomous Region, China) soaked with normal saline warmed to 37°C and applied intraoperatively. 4) Warming of fluid for surgical field flush: the fluid was warmed to 37°C with an electric thermostat. 5) Heating blanket: An Astopad Plus electric blanket (Stihler Electronic, Stuttgart, Germany) was prewarmed and then set to 38.5°C and spread beneath the patient’s body except for the head.

The temperature of the operating room was set at 23°C with humidity at 50%. The temperature sensors for the patient’s nasopharynx and rectum were first connected to a BeneView T6 model vital signs monitor (Shenzhen Mindray Bio-Medical Electronics Co., Ltd., Shenzhen, China). Rectal and nasal probes were then inserted into awake patients. The nasopharyngeal temperature sensor was inserted into the nasopharynx as far as the distance between the inner side of the nasal ala and the earlobe. The rectal temperature sensor was inserted to a depth of more than 6 cm from the anus. The patient’s age, height, and weight; preoperative temperature, heart rate, and blood pressure; fluid volume of the surgical field flush; intraoperative volumes of bleeding, blood transfusion, and fluid infusion; urine volume; and surgeon’s name were recorded. Temperatures in the nasopharynx and rectum were monitored and recorded continuously from the time of patients’ entrance into the operating room until completion of the surgery. Rectal and nasal probes were removed before patients leave the operating room. Nasophayngeal and rectal temperatures are those recorded at the end of surgery as described below.

### Statistical Analysis

Means and standard deviations with range or frequency and percentage were summarized for each variable unless otherwise stated. Rectal and nasopharyngeal temperatures were measured over the study period and analyzed at completion of surgery. Comparisons were performed with analysis of variance (ANOVA) with post hoc comparison adjusted by Tukey’s test. Data were analyzed with SAS 9.0 software (SAS Institute Inc, Cary, NC, USA), and a *P* value <0.05 was considered statistically significant.

## Results

The 160 patients selected for the study were between 18 and 60 years old; they included 82 males and 78 females. In terms of ethnicity, 116 patients were Han Chinese; 30 patients were Uyghurs; and ethnic minorities such as Kazaks included 14 patients. No complications or adverse effects were caused by the warming methods used in the study. Differences in patients’ age, height, and weight; preoperative temperature, heart rate, and blood pressure; volume of fluid used to rinse the surgical field; intraoperative volumes of bleeding, blood transfusion, and fluid infusion; and patients’ urine volume in each group were not considered statistically significant (Tables S1 and S2).

### Postoperative Comparison of Nasopharyngeal Temperatures

**Figure 1:**
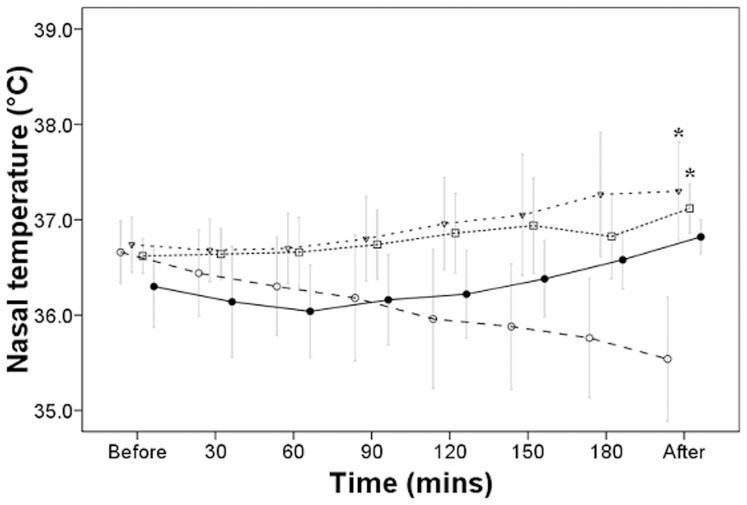
Selected groups of patients’ nasopharyngeal temperatures during surgery. ○ Control group; ▽ Body wraps and heating blanket group; □ Body wraps, heated moist dressing, and heating blanket group; • Heated blood transfusion and fluid infusion, body wraps, heated moist dressing, heated surgical field rinse, and heating blanket group. Mean ± SD, n  = 5, *P<0.05 compared to control group at end of surgery after Tukey’s test.

The mean nasopharyngeal temperature of the group warmed by a combination of body wraps and a heating blanket was 37.3±0.51°C,which was a statistically significantly difference compared to that of control groups A1B1C1D1E1 (Table 1), in which none of the warming methods were used (Table 3), *P*<0.05; this method was the most effective method of patient warming. The second most effective method of warming was a combination of body wraps, heated moist dressings, and a heating blanket. The mean nasopharyngeal temperature of patients in this group was 37.12±0.26°C, which was a statistically significant difference compared to that of control groups A1B1C1D1E1, *P*<0.05 (Figure 1). The third most effective method of warming was a combination of body wraps, heated moist dressings, warming of the surgical rinse fluid, and a heating blanket. The mean nasopharyngeal temperature of patients in this group was 37±0.33°C, which was a statistically significant difference compared to that of control groups A1B1C1D1E1, *P*<0.05 (Table 3). Figure 1 shows the measurement of temperature over time during surgery; the temperatures in the control groups A1B1C1D1E1 dropped slightly over time while temperatures were maintained in the selected groups receiving warming methods.

**Table 3:** Postsurgical nasopharyngeal and rectal temperatures (°C).

Group	Heated transfusion and fluid infusion	Body wraps	Heated moist dressing	Heated surgical filed rinse	Heating blanket	Nasopharyngeal temperature	Rectal temperature
1						35.54±0.65	36.02±0.65
2					Y	36.9±0.37*	37.26±0.26*
3				Y		36.32±0.91	36.62±0.71
4				Y	Y	36.1±0.96	36.5±0.97
5			Y			36.12±0.26	36.36±0.09
6			Y		Y	36.7±0.49	36.96±0.5
7			Y	Y		36.54±0.35	36.44±0.38
8			Y	Y	Y	36.74±0.52	37.06±0.49
9		Y				36.46±0.93	36.76±0.82
10		Y			Y	37.3±0.51*	37.44±0.5*
11		Y		Y		36.86±0.33	36.98±0.35
12		Y		Y	Y	36.92±0.26*	37.1±0.23
13		Y	Y			36.12±0.34	36.36±0.3
14		Y	Y		Y	37.12±0.26*	37.42±0.15*
15		Y	Y	Y		37±0.58*	37.42±0.49*
16		Y	Y	Y	Y	37±0.33*	37.38±0.49*
17	Y					36.44±0.8	36.64±0.77
18	Y				Y	36.78±0.13	37.14±0.09
19	Y			Y		36.02±0.83	36.36±0.74
20	Y			Y	Y	36.94±0.22*	37.32±0.13*
21	Y		Y			36.52±0.88	36.98±0.76
22	Y		Y		Y	36.46±0.39	36.74±0.47
23	Y		Y	Y		36.2±0.35	36.42±0.36
24	Y		Y	Y	Y	36.64±0.51	37.04±0.21
25	Y	Y				36.48±0.36	36.82±0.4
26	Y	Y			Y	36.16±0.58	36.5±0.51
27	Y	Y		Y		36.72±0.51	36.86±0.49
28	Y	Y		Y	Y	36.84±0.47	37.04±0.21
29	Y	Y	Y			36.38±0.38	36.96±0.29
30	Y	Y	Y		Y	36.92±0.42*	37.16±0.53
31	Y	Y	Y	Y		36.3±0.84	36.74±0.73
32	Y	Y	Y	Y	Y	36.82±0.18	37.04±0.21

* *P*<0.05 compared to control group after Tukey’s test.

### Postoperative Comparison of Rectal Temperatures

**Figure 2:**
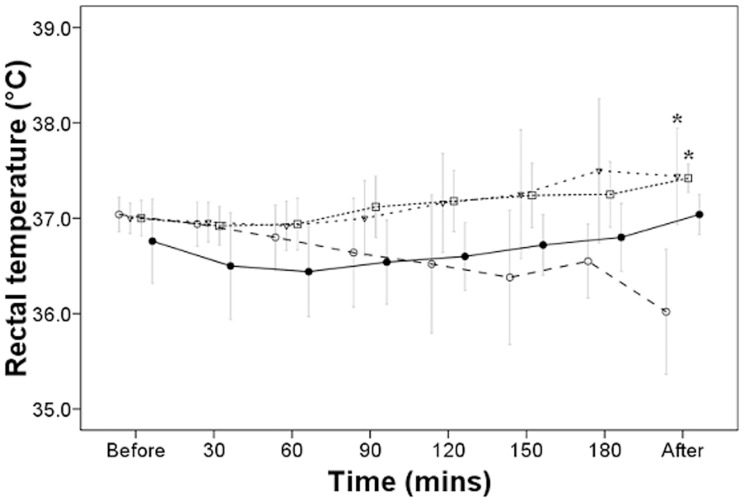
Selected groups of patients’ rectal temperatures during surgery. ○ Control group; ▽ Body wraps and heating blanket group; □ Body wraps, heated moist dressing, and heating blanket group; • Heated blood transfusion and fluid infusion, body wraps, heated moist dressing, heated surgical field rinse, and heating blanket group. Mean ± SD, n  = 5, *P<0.05 compared to control group at end of surgery after Tukey’s test.

The mean rectal temperature of the patient group warmed by a combination of body wraps and a heating blanket was 37.44±0.5°C, which was a statistically significant difference compared to that of control groups A1B1C1D1E1, *P*<0.05. The rectal temperature of patients in the group warmed by a combination of body wraps, heated moist dressings, and a heating blanket was 37.42±0.15°C, which was a statistically significant difference compared to that of control groups A1B1C1D1E1, *P*<0.05. The rectal temperature of patients in the group warmed by a combination of body wraps, heated moist dressings, and warming of the surgical rinse fluid was 37.42±0.49°C, which was statistically significant difference compared to that of control groups A1B1C1D1E1, *P*<0.05. Similar to the record of nasopharyngeal temperatures in Figure 1, Figure 2 shows the rectal temperatures measured over time during surgery. The temperature in control group A1B1C1D1E1 dropped slightly over time while temperatures were maintained in the selected groups receiving warming methods.

## Discussion

In this study, we evaluated the use of low- or no-power, low-cost and readily available alternative warming methods to help maintain normothermia in abdominal surgery patients. When we compared temperatures of abdominal surgery patient groups receiving three specific warming methods with temperatures of control groups not receiving these methods, significant differences were revealed in temperatures maintained during the surgeries between the warmed groups and controls. The three most effective warming methods in order of levels of efficacy in maintaining normothermia were: 1) a combination of body wraps and a heating blanket, 2) a combination of body wraps, heated moist dressings, and a heating blanket, and 3) a combination of body wraps, heated moist dressings, warming of the surgical rinse fluid, and with or without a heating blanket. These methods all use less or no electric power and readily available supplies, and are inexpensive. As such they are practical and applicable for use in certain geographic areas or specific circumstances, including in power outages during natural disasters.

Core temperature can be measured in several ways based on the site of measurement. Oral sublingual temperature and rectal temperature measurements are the two most frequently used methods for measuring core temperature in thermoregulatory investigations [4]. A review of core measurement methods indicated that rectal measurement is considered the gold standard for core measurements, although it is slower to reach temperature [5]. Oral (sublingual) measures were sometimes found to be inaccurate but nasopharyngeal measurements were more reliable. Other measures such as tympanic temperature measurement, bladder and arterial measurements, and esophageal temperature measurement are not convenient and are reserved for specific clinical circumstances; esophageal temperature measurement is not advised for general core measurements because of the difficulty of inserting the thermistor and general patient discomfort. Therefore, for this study, we relied on measurements of nasopharyngeal and rectal temperatures, which are both easily measured and relatively stable.

The value of maintaining normothermia in patients undergoing abdominal surgery under general anesthesia is accepted. Mild hypothermia is recognized when the patient’s core temperature is 35–36.4°C; the intraoperative incidence rate of mild hypothermia is reported to be 50%–70% [6]. Recent studies of patient safety during the perioperative period and the consequent development of relevant research have shown that there are several forms of serious impairment to the human organism when the core temperature of a patient falls below 36°C [7]. A higher incidence of cardiovascular complications is caused by hypothermia, including angina, myocardial infarction, and cardiac arrest [8]. Hypothermia inhibits blood coagulation, which may increase intraoperative blood loss. Blood loss in turn may lead to a higher rate of postoperative complications [9]. In addition, hypothermia has an impact on the immune system, which markedly increases the incidence of wound infections [10], extended hospitalization, and a worsening of the patient’s prognosis [11]. A decrease in core body temperature slows the metabolism of anesthetic agents, which in turn delays the patient’s recovery from general anesthesia [12]. Since hypothermia is known to increase susceptibility to complications [13], the higher risk of complications during the perioperative period, the higher rate of postoperative complications, and a prolonged stay in the hospital may lead to increases in the patient’s financial burden [7]. However, extending the warming methods to the perioperative period can provide beneficial effects that may also be preventative in the sense of reducing risks, and at only minimal additional cost [13].

Hypothermia during abdominal surgery is the result of heat loss through radiation, convection, conduction, and evaporation. The operating room in our hospital is equipped with a clean laminar airflow, with room temperature set at 23°C and humidity at 50%. It is a cool environment for abdominal surgery patients, and this factor increases the loss of body heat that causes hypothermia. The use of a disinfectant fluid at low temperature prior to the operation leads to loss of body heat through conduction and evaporation. General anesthesia depresses the mechanisms of body thermoregulation; thus a lengthy abdominal surgical procedure that exposes the internal organs induces hypothermia. A study of the effects of intravenous fluid warming and skin surface warming for elderly patients undergoing abdominal surgery demonstrated that skin surface warming was more effective in preventing hypothermia and maintaining acid-base balance during administration of general anesthesia, although it didn’t sustain normal body temperatures in these elderly patients [14].

**Table 4:** The marginal effects of each factor is demonstrated for nasopharyngeal temperature and rectal temperature.

	Nasopharyngeal temperature, p value	Rectal temperature, p value
**Heated transfusion and fluid infusion**	0.445	0.814
**Body wraps**	0.003	0.003
**Heated moist dressing**	0.585	0.410
**Heated surgical filed rinse**	0.288	0.556
**Heating blanket**	<0.0001	<0.0001

Unintentional hypothermia occurs commonly during surgery and measures are introduced to prevent heat loss. To compensate for inadvertent hypothermia, operating rooms usually introduce some method of warming patients, including warming blankets with circulating water, air insulation blankets, or warmed fluid to maintain patients’ body temperature. It is also possible, however, to work out the best methods for maintaining body temperature using traditional warming techniques instead of advanced equipment for general surgical patients in certain locations–such as the author’s present hospital–that are under financial constraints. The low-cost method evaluated in this study are ideal for institutions with financial constraints; these methods use materials that are readily available in almost all operating rooms and that are easily applied, which makes them practical and applicable in circumstances in which technology-intensive methods are not available. The results of this research demonstrate that the combined use of warming measures contributes to the best outcome for patients. The loss of heat generated inside the body occurs primarily through exposure of the body surface to the surrounding environment. Since the greater portion of an abdominal surgery patient’s body is exposed in a low-temperature operating room, the greatest the amount of heat lost is via the skin. Wrapping the body surface except for the sterile surgical field to reduce loss of body heat via radiation, and obstructing immediate contact between the skin and the cold environment is effective in minimizing intraoperative heat loss. The same rationale applies to the use of a heating blanket; however, a heating blanket is an active heat preserving measure that provides an environment with a relatively higher temperature than the patient’s body. Heating blankets are effective in preventing loss of body heat and elevating body temperature by reducing heat loss through the skin via radiation and conduction. They even reverse the processes of radiation, conduction, and convection through the flow of heat from the blanket to the contacted skin surface. Recently, thermal suits constructed of special heat-preserving textiles were shown to be a good alternative to conventional warming measures in reducing heat loss in surgical procedures using regional anesthesia, although conventional methods such as heating blankets and warmed intravenous fluids were used simultaneously [15]. Although the thermal suits did not entirely prevent decreases in patients’ body temperatures, they did so to a greater extent than in controls who received the conventional methods only. The low temperature of wet dressings is another leading contributor to intraoperative hyothermia, although moist dressings soaked with normal saline at a temperature of 37°C have the effect of a certain amount of thermal warming. Using surgical field rinse warmed to 37°C reduces the gap in temperature between the abdominal organs and the fluid, whose temperature may even be higher than that of the abdominal cavity and, as such, may help to maintain body temperature by minimizing the effects of radiation and convection between the exposed organs and the environment.

The limitation of this study is that we have compared all 32 individual groups and it wasn’t powered for this approach. The proper analysis for factorial approach is to evaluate the marginal effects of each factor. From the analysis for the marginal effects of each factor (Table 4), body wraps and heating blanket are the two major factors having marginal effects. The analysis result is well fit in our conclusion.

### Conclusion

We conclude that the use of a combination of body wrapping and a heating blanket is the most effective method of warming abdominal surgery patients to ensure successful anesthesia and surgery, and to reduce the risk of postoperative complications. The second most effective method is a combination of body wrapping, heated moist dressings, and a heating blanket. The third best method is a combination of body wrapping, heated moist dressings, and warmed surgical rinse fluid, with or without a heating blanket. These methods are practically applicable when low-cost methods are indeed needed.

## Supporting Information

Table S1 **General preoperative data for each group.**
(DOC)

Table S2
**Intraoperative conditions of each group (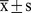).**
(DOC)
