# Supplementary material for: Methods of Patient Warming during Abdominal Surgery
Source: PLoS One. 2012 Jul 11;7(7):e39622. doi: 10.1371/journal.pone.0039622 (PMC3394771; doi:10.1371/journal.pone.0039622)
Supplement: Table S1 — General preoperative data for each group. (DOC) [file pone.0039622.s001.doc]

Supplementary tables

Table S1. General preoperative data for each group.

| **Group** | **Patient Age** | **Weight**  **(kg)** | **Pre-OP Temp (°C)** | **Heart rate** | **Systolic blood pressure** | **Diastolic blood pressure** | **Anesthesia duration** | **Surgery duration** |
| --- | --- | --- | --- | --- | --- | --- | --- | --- |
| 1 | 49.2±8.7 | 64.2±12.7 | 36.4±0.1 | 87.4±19.8 | 112.8±13.0 | 79.0±7.4 | 234.8±34.5 | 207.8±33.0 |
| 2 | 43.4±18.4 | 66.6±6.5 | 36.6±0.2 | 79.2±7.7 | 117.6±26.4 | 69.4±8.2 | 245.4±62.6 | 215.6±54.5 |
| 3 | 49.4±7.1 | 69.6±11.2 | 36.6±0.1 | 77.0±10.1 | 130.0±14.1 | 85.0±11.2 * | 298.8±80.0 | 239.8±68.0 |
| 4 | 45.8±14.4 | 62.4±9.6 | 36.6±0.2 | 77.6±9.5 | 113.0±12.0 | 69.0±7.4 | 251.6±37.7 | 217.6±29.0 |
| 5 | 40.4±14.3 | 61.2±8.6 | 36.5±0.1 | 81.6±2.6 | 123.0±17.9 | 71.0±7.4 | 253.2±67.9 | 222.6±62.8 |
| 6 | 48.2±7.8 | 60.6±14.8 | 36.6±0.2 | 77.6±6.8 | 126.0±26.1 | 82.4±18.1 | 303.6±53.1 | 264.8±50.4 |
| 7 | 50.0±11.2 | 64.6±4.4 | 36.4±0.1 | 79.0±3.7 | 126.0±21.9 | 75.0±10.0 | 219.8±52.0 | 191.6±45.6 |
| 8 | 46.2±13.4 | 62.6±10.7 | 36.5±0.1 | 81.4±7.5 | 119.4±16.9 | 71.6±11.4 | 261.6±48.5 | 232.8±43.2 |
| 9 | 46.2±16.9 | 62.4±21.6 | 36.5±0.2 | 88.4±11.0 | 114.8±9.8 | 73.8±5.2 | 232.4±60.4 | 198.4±49.7 |
| 10 | 49.4±7.8 | 69.2±18.3 | 36.5±0.2 | 76.0±6.8 | 117.4±17.9 | 73.0±11.0 | 225.2±52.2 | 194.2±59.4 |
| 11 | 46.8±5.4 | 62.8±12.1 | 36.7±0.2 | 82.0±2.0 | 123.4±6.1 | 72.0±4.5 | 208.6±57.8 | 164.6±54.9 |
| 12 | 45.8±11.1 | 69.0±20.5 | 36.5±0.3 | 86.4±19.1 | 110.0±12.2 | 68.0±11.0 | 201.6±42.3 | 176.6±49.8 |
| 13 | 50.4±14.4 | 66.4±10.6 | 36.4±0.2 | 70.0±12.1 | 125.4±16.0 | 77.2±14.8 | 250.0±59.5 | 227.0±53.1 |
| 14 | 35.6±6.8 | 66.2±19.8 | 36.4±0.1 | 80.6±5.8 | 116.4±11.2 | 78.0±8.4 | 259.6±57.4 | 230.0±61.8 |
| 15 | 47.0±12.6 | 60.4±19.3 | 36.5±0.1 | 72.2±9.8 | 115.4±14.5 | 72.6±8.7 | 263.0±56.9 | 225.4±44.6 |
| 16 | 49.0±6.8 | 68.0±7.8 | 36.5±0.2 | 78.0±3.5 | 120.8±20.3 | 70.8±8.6 | 271.2±53.0 | 231.0±49.3 |
| 17 | 47.4±13.0 | 70.0±7.5 | 36.5±0.2 | 77.6±11.3 | 137.6±5.5 | 89.0±5.3 * | 264.0±48.4 | 228.0±46.2 |
| 18 | 55.4±6.2 | 64.4±12.6 | 36.4±0.1 | 80.8±1.8 | 132.0±14.8 | 75.0±5.0 | 197.0±26.6 | 173.0±25.0 |
| 19 | 51.6±5.1 | 67.4±14.8 | 36.7±0.3 | 96.8±23.3 | 122.4±16.8 | 72.8±7.0 | 220.0±88.6 | 179.4±71.4 |
| 20 | 46.0±7.7 | 59.2±8.6 | 36.6±0.2 | 85.2±4.4 | 121.4±12.4 | 71.6±9.8 | 279.4±74.8 | 244.2±73.5 |
| 21 | 46.6±13.6 | 63.0±6.3 | 36.7±0.2 | 72.6±5.3 | 108.8±12.5 | 66.8±11.9 | 222.2±85.9 | 198.8±78.6 |
| 22 | 40.2±14.1 | 64.4±13.0 | 36.5±0.1 | 77.6±8.0 | 121.0±10.2 | 75.2±10.3 | 247.2±98.5 | 215.4±75.6 |
| 23 | 48.0±11.0 | 65.2±10.0 | 36.7±0.2 | 82.8±6.9 | 127.6±20.9 | 76.4±10.7 | 210.2±29.5 | 174.8±31.3 |
| 24 | 52.6±5.1 | 75.6±11.3 | 36.6±0.3 | 79.4±7.0 | 121.4±12.6 | 74.0±11.4 | 279.4±37.7 | 244.4±48.8 |
| 25 | 48.6±8.7 | 64.4±7.4 | 36.6±0.1 | 81.2±7.0 | 120.0±14.1 | 76.0±8.9 | 217.2±80.4 | 188.4±73.3 |
| 26 | 47.0±10.0 | 69.6±11.2 | 36.4±0.2 | 80.6±9.2 | 116.2±11.5 | 69.4±9.3 | 249.6±80.0 | 215.6±67.0 |
| 27 | 48.2±13.9 | 59.0±11.8 | 36.5±0.2 | 84.4±9.2 | 106.4±11.2 | 59.8±0.4 | 275.0±61.6 | 234.4±59.6 |
| 28 | 50.4±8.3 | 66.0±21.1 | 36.7±0.3 | 76.8±13.0 | 116.4±8.6 | 70.8±10.6 | 254.0±32.2 | 215.6±47.2 |
| 29 | 46.4±12.5 | 67.6±14.4 | 36.4±0.2 | 81.0±9.1 | 126.0±8.9 | 78.0±7.6 | 192.0±29.0 | 167.6±31.1 |
| 30 | 50.6±16.7 | 68.2±14.4 | 36.5±0.3 | 79.6±8.3 | 124.0±15.2 | 75.0±8.7 | 251.4±74.4 | 223.4±67.4 |
| 31 | 54.2±4.8 | 71.3±20.9 | 36.3±0.2 | 87.2±12.0 | 131.0±16.7 | 77.0±13.0 | 306.2±46.7 | 271.8±33.3 |
| 32 | 55.2±6.1 | 63.4±9.5 | 36.5±0.2 | 76.0±8.7 | 118.6±18.2 | 73.0±11.0 | 284.8±50.6 | 242.4±46.9 |

*P<0.05, significantly different from other groups after Tukey’s adjustment
