# Supplementary material for: Methods of Patient Warming during Abdominal Surgery
Source: PLoS One. 2012 Jul 11;7(7):e39622. doi: 10.1371/journal.pone.0039622 (PMC3394771; doi:10.1371/journal.pone.0039622)
Supplement: Table S2 — Intraoperative conditions of each group (). (DOC) [file pone.0039622.s002.doc]

Table S2.Intraoperative conditions of each group （$\overline{\text{x}}\pm\text{s}$）

| **Group** | **Volume of surgical field rinsing (mL)** | **Intraoperative bleeding volume (mL)** | **Intraoperative volume of fluid infusion (mL)** | **Number of saline pads** | **Number of large gauze pads** | **Urine volume (mL)** |
| --- | --- | --- | --- | --- | --- | --- |
| 1 | 2500±520 | 244±80 | 2040±288 | 1±0 | 15.4±4.3 | 443±325 |
| 2 | 2870±1264 | 288±194 | 2240±775 | 1±0.7 | 15.4±12.1 | 440±292 |
| 3 | 3890±2736 | 1313±1872 | 3320±2313 | 4±4.2* | 30±34.4 | 444±317 |
| 4 | 2860±647 | 215±147 | 1800±405 | 1.2±0.4 | 10.6±3.8 | 388±233 |
| 5 | 2500±1414 | 280±164 | 1800±888 | 1.4±0.5 | 15.4±11.3 | 476±257 |
| 6 | 2700±1110 | 400±139 | 3000±1076 | 1.6±0.5 | 15.4±6.3 | 664±629 |
| 7 | 2890±827 | 278±121 | 1690±524 | 1.8±1.1 | 11.4±3.4 | 300±94 |
| 8 | 3130±1023 | 269±152 | 2230±531 | 1.2±1.1 | 15±4.9 | 360±165 |
| 9 | 1620±658 | 201±128 | 1460±230 | 0.8±0.4 | 11±2.9 | 485±306 |
| 10 | 2050±620 | 247±211 | 1750±707 | 0.8±0.4 | 10.6±1.3 | 310±171 |
| 11 | 2490±849 | 203±133 | 1540±270 | 0.8±0.4 | 11±2.4 | 314±109 |
| 12 | 1760±477 | 279±156 | 1770±609 | 1±0.7 | 9.8±2 | 200±77 |
| 13 | 2910±1338 | 334±115 | 1670±385 | 0.8±0.4 | 14.2±5.6 | 600±274 |
| 14 | 2270±667 | 240±148 | 1560±360 | 1.6±1.1 | 10.6±2.4 | 458±371 |
| 15 | 2240±411 | 296±188 | 2220±1026 | 1±0 | 10.6±4 | 256±120 |
| 16 | 3100±822 | 530±233 | 2120±475 | 1.8±1.1 | 20±9.5 | 298±171 |
| 17 | 2830±622 | 668±925 | 2170±936 | 2±1.2 | 13.8±7.6 | 714±479 |
| 18 | 2200±948 | 143±77 | 1590±282 | 0.8±0.4 | 10.8±3 | 366±227 |
| 19 | 2820±1491 | 995±1383 | 2510±1798 | 1.4±1.1 | 18.6±19 | 532±766 |
| 20 | 2550±787 | 500±324 | 2310±1027 | 1.2±0.4 | 18.8±12.9 | 454±210 |
| 21 | 1660±645 | 324±305 | 1620±503 | 1.6±0.5 | 13.8±7.5 | 344±190 |
| 22 | 4200±3276 | 495±566 | 2140±932 | 1.4±0.5 | 16.2±15 | 504±400 |
| 23 | 1840±241 | 170±71 | 1880±264 | 1±0.7 | 11.6±5 | 350±194 |
| 24 | 3020±892 | 520±463 | 2210±614 | 1.4±0.9 | 9.8±2 | 346±182 |
| 25 | 2080±704 | 220±115 | 2015±564 | 1.2±0.4 | 10.6±6.7 | 476±324 |
| 26 | 3150±1810 | 386±279 | 1730±704 | 1.2±0.8 | 16.4±11.3 | 236±152 |
| 27 | 2590±1227 | 891±1189 | 2660±637 | 1±0 | 13.6±4 | 548±376 |
| 28 | 3370±1592 | 198±69 | 1900±379 | 1.2±0.4 | 15.2±3.1 | 293±100 |
| 29 | 1930±954 | 216±139 | 1810±561 | 0.6±0.5 | 8.8±1.6 | 420±315 |
| 30 | 2540±1064 | 462±333 | 2430±805 | 0.8±0.4 | 14.6±6.5 | 370±280 |
| 31 | 3130±1417 | 879±1135 | 3210±1172 | 1.4±0.9 | 26±22.5 | 796±529 |
| 32 | 2320±702 | 394±85 | 2015±601 | 1.4±0.5 | 12.4±1.7 | 454±26 |

*P<0.05, significantly different from other groups after Tukey’s adjustment
